# Editorial: Obstructive Sleep Apnea and the Brain

**DOI:** 10.3389/fsurg.2018.00078

**Published:** 2018-12-21

**Authors:** Haralampos Gouveris, Danny J. Eckert

**Affiliations:** ^1^Department of Otorhinolaryngology, Medical Centre of the Johannes Gutenberg University, Mainz, Germany; ^2^Neuroscience Research Australia (NeuRA) and the University of New South Wales, Sydney, NSW, Australia

**Keywords:** sleep apnea, sleep disruption, brain, multiple sclerosis, neurobehavioural, stroke, insomnia, female

There is accumulating evidence that sleep disturbance, including sleep disorders such as obstructive sleep apnoea, can adversely affect restorative brain processes and function (1–5). In this interdisciplinary Research Topic, the interplay between clinical diseases of the central nervous system (CNS) such as multiple sclerosis Hensen et al. and stroke Stevens et al. and sleep disordered breathing is highlighted. Both of these reviews discuss potential shared mechanisms, consequences, and novel treatment approaches. In addition, a review of the clinical challenges of managing patients with comorbid insomnia and obstructive sleep apnoea and treatment modalities, including the role of cognitive behavioural therapy, is presented Bahr et al.. In the final piece in this series, original research findings provide insight into a novel computerised test battery to examine neurobehavioural function in adults with and without obstructive sleep apnoea D'Rozario et al..

From a mechanistic perspective, the coining of the concept of at least four phenotypic traits that cause sleep disordered breathing has recently emerged (6). Namely, high loop gain, low respiratory arousal threshold, poor pharyngeal muscle responsiveness during sleep, and upper airway anatomic compromise (6–8). This new framework has provided insights into this common condition. Clinical phenotypes to define the different clinical manifestations and consequences of sleep disordered breathing have also recently been described (9). These mechanistic and clinical concepts pave the way for targeted therapy or “precision sleep medicine” to reduce the adverse health, safety, and social impact of untreated sleep disordered breathing (10). Indeed, the framework to provide personalised therapy for sleep disordered breathing in those without major comorbid disease has been set (11). Non-CPAP interventions that show promise in small physiological studies or clinical trials include pharmacologic, surgical, oral appliance, positional, oxygen, myofunctional training, or combinations thereof (11).

In accordance with these concepts, Hensen et al. detailed review highlights the differences in clinical presentation of sleep disordered breathing in people with MS. While the prevalence remains unclear, the available evidence suggests that sleep disordered breathing is likely at least as common in people with MS if not more so. This is despite clear differences in the clinical manifestation of sleep disordered breathing in this patient population that would tend to favour lower risk of sleep apnoea (i.e., predominantly non-obese females). This suggests that the causes, and by extension the ideal therapy or therapies for sleep apnoea, is likely to be quite different in people with MS compared to typical sleep apnoea patient populations. Concepts of bidirectionality between the causes and consequences of sleep disordered breathing and MS (e.g. inflammation, hypoxia, depression, fatigue/sleepiness, and neurocognitive impairment) are also proposed. These new concepts pave the way for an important research agenda that has the potential to lead to targeted therapies to reduce the common consequences of both conditions and potentially MS disease progression.

Another example of CNS disruption and high rates of sleep disruption/sleep apnoea is stroke. Neuroimaging studies after acute neurologic impairment (e.g., in the acute phase after stroke) combined with sleep investigations may provide important insight into the central nervous system mechanisms of sleep disordered breathing. In their mini review, Stevens et al. discuss the need for new knowledge into the specific causes of sleep disordered breathing post stroke using mechanistic phenotyping concepts. Similar to MS, the size and location of CNS lesions is likely crucial in determining the type and extent to sleep disordered breathing that occurs post stroke. Despite evidence that functional recovery outcomes post stroke improve after treatment of sleep disordered breathing (12, 13), CPAP therapy is particularly poorly tolerated in this patient population. Thus, there is a need to test and develop new therapeutic approaches for sleep disordered breathing to improve post stroke outcomes Stevens et al.. The same may apply to other disorders that affect central locomotor control such as Parkinson's disease (14–16).

Bahr et al. highlight the treatment challenges when two common unwanted bedfellows co-exist, namely insomnia and sleep apnoea. It is clear that when insomnia, which is characterised by cortical hyperarousal, combines with sleep disordered breathing there are additive or potentially synergistic adverse consequences (17–19). The goal remains to better understand the specific causes of each and different phenotypic presentations so that therapies can be tailored accordingly.

In the final original research contribution in this series, D' Rozario et al. highlight the shortcomings of sleep study parameters using gold standard polysomnography to accurately identify neurobehavioural dysfunction. Indeed, there is currently no standardised neuropsychological test battery to assess daytime dysfunction which can be administered in a timely manner in a sleep laboratory setting. The 30 min computerised test battery that they developed was used to examine neurobehavioural function in over 200 people with untreated sleep apnoea versus a control group of 50 healthy participants. People with OSA had impaired neurobehavioural performance in all domains. Consistent with previous findings, polysomnography disease severity measures were only weakly associated with performance. The authors conclude that the test battery provides a sensitive method of assessing daytime dysfunction in patients with sleep apnoea. Nonetheless, there remains a need to further test and develop instruments to assess cognition, attention, alertness, short-term and long- term memory and executive function in the context of sleep disruption (20–23). There is increasing evidence that a possible gender—specific dimension of sleep disordered breathing should also be taken into account (20, 24–26).

In summary, there may be important bidirectional relationships between sleep disruption/sleep disordered breathing and a range of disorders that adversely affect brain/CNS function. Sophisticated approaches to better understand these links are required along with simple, accurate testing tools that can be used in the clinical setting to inform treatment decision making. This will then facilitate targeted strategies to improve sleep disruption/sleep disordered breathing across a range of CNS/brain disorders. This approach has the potential to also reduce the numerous shared disease consequences which often coexist in people who have sleep disordered breathing and a CNS/brain disorder. Given the increasing rates of sleep apnoea and disorders of the CNS/brain and considerable gaps in knowledge and treatment, these topics remain research priorities.

## Author Contributions

Both HG and DE drafted and revised the manuscript and gave approval for publication of this manuscript.

### Conflict of Interest Statement

DE has received research grants from Bayer, Apnimed and has a Cooperative Research Centre (CRC)-P grant, a collaboration between the Australian Government, Academia and Industry (Industry partner Oventus Medical). The remaining author declares that the research was conducted in the absence of any commercial or financial relationships that could be construed as a potential conflict of interest.

## References

[1] Ayalon L Ancoli-Israel S Drummond SP. Obstructive sleep apnea and age: a double insult to brain function? Am J Respir Crit Care Med. (2010) 182:413–9. 10.1164/rccm.200912-1805OC 20395556 PMC2921601

[2] Canessa N Castronovo V Cappa SF Aloia MS Marelli S Falini A. Obstructive sleep apnea: brain structural changes and neurocognitive function before and after treatment. Am J Respir Crit Care Med. (2011) 183:1419–26. 10.1164/rccm.201005-0693OC 21037021

[3] Olaithe M Bucks RS. Executive dysfunction in OSA before and after treatment: a meta-analysis. Sleep (2013) 36:1297–305. 10.5665/sleep.2950 23997362 PMC3738038

[4] Jackson ML McEvoy RD Banks S Barnes M. Neurobehavioral impairment and CPAP treatment response in mild-moderate obstructive sleep apneas. J Clin Sleep Med. (2018) 14:47–56. 10.5664/jcsm.6878 29198304 PMC5734892

[5] Song X Roy B Kang DW Aysola RS Macey PM Woo MA. Altered resting-state hippocampal and caudate functional networks in patients with obstructive sleep apnea. Brain Behav. (2018) 8:e00994. 10.1002/brb3.994 29749715 PMC5991585

[6] Eckert DJ White DP Jordan AS Malhotra A Wellman A. Defining phenotypic causes of obstructive sleep apnea. Identification of novel therapeutic targets. Am J Respir Crit Care Med. (2013) 188:996–1004. 10.1164/rccm.201303-0448OC 23721582 PMC3826282

[7] Edwards BA Eckert DJ McSharry DG Sands SA Desai A Kehlmann G. Clinical predictors of the respiratory arousal threshold in patients with obstructive sleep apnea. Am J Respir Crit Care Med. (2014) 190:1293–300. 10.1164/rccm.201404-0718OC 25321848 PMC4315811

[8] Owens RL Edwards BA Eckert DJ Jordan AS Sands SA Malhotra A. An integrative model of physiological traits can be used to predict obstructive sleep apnea and response to non positive airway pressure therapy. Sleep (2015) 38:961–70. 10.5665/sleep.4750 25515107 PMC4434563

[9] Keenan BT Kim J Singh B Bittencourt L Chen NH Cistulli PA. Recognizable clinical subtypes of obstructive sleep apnea across international sleep centers: a cluster analysis. Sleep (2018) 41:214. 10.1093/sleep/zsx214 29315434 PMC5914381

[10] Hillman D Mitchell S Streatfeild J Burns C Bruck D Pezzullo L. The economic cost of inadequate sleep. Sleep (2018) 41:83. 10.1093/sleep/zsy083 29868785

[11] Carberry JC Amatoury J Eckert DJ. Personalized management approach for OSA. Chest (2018) 153:744–55. 10.1016/j.chest.2017.06.011 28629917

[12] Ryan CM Bayley M Green R Murray BJ Bradley TD. Influence of continuous positive airway pressure on outcomes of rehabilitation in stroke patients with obstructive sleep apnea. Stroke (2011) 42:1062–7. 10.1161/STROKEAHA.110.597468 21372306

[13] Bravata DM Sico J Vaz Fragoso CA Miech EJ Matthias MS Lampert R. Diagnosing and treating sleep apnea in patients with acute cerebrovascular disease. J Am Heart Assoc. (2018) 7:e008841. 10.1161/JAHA.118.008841 30369321 PMC6201384

[14] Valko PO Hauser S Sommerauer M Werth E Baumann CR. Observations on sleep-disordered breathing in idiopathic Parkinson's disease. PLoS ONE (2014) 9:e100828. 10.1371/journal.pone.0100828 24968233 PMC4072709

[15] Harmell AL Neikrug AB Palmer BW Avanzino JA Liu L Maglione JE. Obstructive sleep apnea and cognition in Parkinson's disease. Sleep Med. (2016) 21:28–34. 10.1016/j.sleep.2016.01.001 27448468 PMC4963611

[16] Mery VP Gros P Lafontaine AL Robinson A Benedetti A Kimoff RJ. Reduced cognitive function in patients with Parkinson disease and obstructive sleep apnea. Neurology (2017) 88:1120–8. 10.1212/WNL.0000000000003738 28228566

[17] Gupta MA Knapp K. Cardiovascular and psychiatric morbidity in obstructive sleep apnea (OSA) with insomnia (sleep apnea plus) versus obstructive sleep apnea without insomnia: a case-control study from a Nationally Representative US sample. PLoS ONE (2014) 9:e90021. 10.1371/journal.pone.0090021 24599301 PMC3943798

[18] Lang CJ Appleton SL Vakulin A McEvoy RD Wittert GA Martin SA. Co-morbid OSA and insomnia increases depression prevalence and severity in men. Respirology (2017) 22:1407–15. 10.1111/resp.13064 28589663

[19] Cho YW Kim KT Moon HJ Korostyshevskiy VR Motamedi GK Yang KI. Comorbid insomnia with obstructive sleep apnea: clinical characteristics and risk factors. J Clin Sleep Med. (2018) 14:409–17. 10.5664/jcsm.6988 29458695 PMC5837842

[20] Yaffe K Laffan AM Harrison SL Redline S Spira AP Ensrud KE. Sleep-disordered breathing, hypoxia, and risk of mild cognitive impairment and dementia in older women. JAMA (2011) 306:613–9. 10.1001/jama.2011.1115 21828324 PMC3600944

[21] Sharma RA Varga AW Bubu OM Pirraglia E Kam K Parekh A. Obstructive sleep apnea severity affects amyloid burden in cognitively normal elderly. a longitudinal study. Am J Respir Crit Care Med. (2018) 197:933–43. 10.1164/rccm.201704-0704OC 29125327 PMC6020410

[22] Gosselin N Baril AA Osorio RS Kaminska M Carrier J. Obstructive sleep apnea and the risk of cognitive decline in older adults. Am J Respir Crit Care Med. (2018) [Epub ahead of print]. 10.1164/rccm.201801-0204PP 30113864 PMC6943882

[23] Gagnon K Baril AA Montplaisir J Carrier J Chami S Gauthier S Detection of mild cognitive impairment in middle-aged and older adults with obstructive sleep apnea. Eur Respir J. (2018) 2018:1801137 10.1183/13993003.01137-2018 30190270

[24] Ramos AR Tarraf W Rundek T Redline S Wohlgemuth WK Loredo JS. Obstructive sleep apnea and neurocognitive function in a Hispanic/Latino population. Neurology (2015) 84:391–8. 10.1212/WNL.0000000000001181 25540308 PMC4336004

[25] Roca GQ Redline S Claggett B Bello N Ballantyne CM Solomon SD. Sex-specific association of sleep apnea severity with subclinical myocardial injury, ventricular hypertrophy, and heart failure risk in a community-dwelling cohort: the atherosclerosis risk in communities-sleep heart health study. Circulation (2015) 132:1329–37. 10.1161/CIRCULATIONAHA.115.016985 26316620 PMC4596785

[26] Gouveris H Bahr K Jahn C Matthias C Simon P. The apnea-hypopnea index underestimates systemic inflammation in women with sleep-disordered breathing. J Womens Health (2018) 27:920–6. 10.1089/jwh.2017.6819 29630436

